# Case Report: Penile malignant melanoma: insights from a three-case series and literature review on diagnosis and management

**DOI:** 10.3389/fonc.2026.1817540

**Published:** 2026-07-17

**Authors:** Xiaoxiao Xu, Xiaolong Hou, Hongze Li, Yiming Chen, Bohao Jiang, Hao Zhang

**Affiliations:** Department of Urology, The First Hospital of China Medical University, Shenyang, China

**Keywords:** case report, melanoma, penile melanoma, review, treatment

## Abstract

**Background:**

Penile melanoma (PM) is an exceptionally rare and aggressive mucosal melanoma. Currently, it remains poorly understood, and no dedicated clinical guidelines have been established. This study aimed to characterize its clinical features and summarize its treatment options through our original case reports and a contemporary literature review.

**Case presentation:**

We present three cases of our medical center, all of whom underwent surgical treatment and were pathologically diagnosed with primary penile melanoma. The first case also underwent postoperative genetic testing and subsequent chemotherapy and achieved a marked reduction of lymph node metastasis. In terms of prognosis, two of them died of progression and one of other diseases. Our literature review subsequently analyzed 27 additional reported cases of PM, which mainly affects middle-aged and older men, with typical manifestations being pigmented patches or nodules on the glans or foreskin. Surgical treatment (local or partial penectomy) was the primary option. Adjuvant therapies, such as chemotherapy and radiotherapy, are commonly used to prevent recurrence, and emerging novel treatments are being evaluated in clinical trials, including immune checkpoint inhibitors and targeted therapies targeting genes such as KIT.

**Conclusion:**

Penile melanoma, as a rare but threatening malignancy, requires a personalized management strategy. A reasonable surgical plan should be made to ensure complete resection, with growing evidence supporting the integration of systemic therapies. Further case reports and clinical research are essential to establish standardized protocols.

## 1 Introduction

Melanoma is one of the deadliest malignant tumors, primarily occurring on the skin’s surface. Non-skin melanoma can occur at multiple sites, including the gastrointestinal and genitourinary tracts and the mucous membranes of the head and neck (1, 2). The incidence of penile melanoma (PM) is extremely low, accounting for less than 2% of all penile tumors and an even lower proportion of less than 0.1% in all melanomas (2), and is associated with high mortality rates and late diagnosis (3). It was first described by Murchison in 1859 (4), but despite its rarity, a limited understanding of its optimal management persists, and no specific treatment guidelines currently exist (5–7).

This paper presents clinical management experiences from three cases of primary malignant melanoma of the penis, provides detailed radiology, pathology, and images of excised specimens, and reviews the literature published over the past decade. By integrating our clinical experience with contemporary published data, we aim to enhance the understanding of its characteristics and management trends, thereby providing a foundation for establishing future decision-making consensus.

## 2 Case presentation

### 2.1 Case 1

The first patient was a 46-year-old male who presented to our hospital with a chief complaint of penile head enlargement lasting one month. The patient denies any chronic diseases and personal/family history of melanoma or other skin diseases. Upon specialized physical examination, the patient was found to have phimosis and a mass measuring approximately 2.0 × 2.0 cm located on the penile glans. No foul odor or ulceration is present. Multiple enlarged lymph nodes are palpable in both inguinal regions, without tenderness. No suspicious lesions were found on the remaining skin and mucous membranes.

Subsequently, the patient underwent a series of examinations at our hospital. Inguinal and penile ultrasound showed a moderately echogenic mass visible anteriorly and to the left of the penis, measuring approximately 3.54 × 2.42 × 2.05cm with a poorly defined border and abundant blood flow. Multiple lymph node echoes are visible in both inguinal regions, with the largest measuring approximately 0.72 × 0.35cm in the right and 2.89 × 2.72cm in the left. They appear round with relatively low echogenicity and show linear blood flow. To further clarify the nature, an abdominal contrast-enhanced CT scan showed a soft tissue mass with ill-defined borders in the glans penis. In the non-contrast phase, it demonstrates a CT value of approximately 46 HU. Following contrast administration, it exhibits heterogeneous enhancement with a CT value of approximately 86 HU. Multiple enlarged superficial lymph nodes are visible bilaterally in the inguinal regions with partial fusion, among which the largest is located in the left inguinal region, measuring approximately 3.2 x 2.7cm. It demonstrates heterogeneous enhancement on contrast-enhanced imaging, with areas of non-enhancing necrosis visible within some lymph nodes, and no involvement of pelvic lymph nodes was observed. A diagnosis of penile malignant tumor with probable lymph node metastasis was made (**Supplementary Figure 1**).

We performed a partial penile resection on the patient following discussion, and bilateral inguinal lymph node dissection was preliminarily planned to be conducted one month later. The surgery aims to achieve complete tumor resection while ensuring negative margins to preserve the quality of life. The glans penis presented with a phimosis-like appearance. After positioning supine, a 2 cm incision was made along the dorsal foreskin, revealing extensive black cauliflower-like growths centered around the urethral meatus, which were suggested to be malignant melanoma by intraoperative pathology (**Figure 1**). A circular incision was made at least 3cm above the diseased tissue, and a ligation of the deep dorsal penile artery was performed. The corpus cavernosum was incised down to the surface of the tunica albuginea of the corpus spongiosum. Approximately 1cm of urethra was dissected free toward the external urethral orifice, transected, and the diseased portion of the penis was removed. The corpus cavernosum, fascial layer, subcutaneous tissue, and skin were sutured sequentially. The urethra was everted to reconstruct the external urethral orifice, and a urinary catheter was finally placed. The patient was discharged on the fifth day postoperatively.

**Figure 1:**
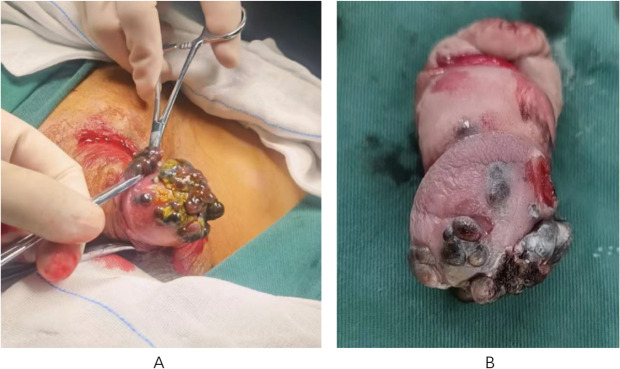
Multiple nodular melanomas of the glans (Case 1). **(A)** The dorsal prepuce was cut open to reveal the glans, which showed extensive black vegetable mass centered on the urethral opening, with almost no normal glans shape. **(B)** The excised specimen of the penis and melanoma during surgery.

The postoperative histopathological diagnosis was confirmed as malignant melanoma, and the immunohistochemical findings supported this diagnosis: CK(±), Vimentin(+), HMB-45(+), Melan A(+), S-100(+), SOX-10(+), Ki-67(>30%) (**Figure 2**). Given the rarity of the disease, genetic testing is performed: KRAS(-), NRAS(-), PIK3CA(-), BRAF(-). The patient declined further bilateral inguinal lymph node biopsy and dissection and underwent intravenous chemotherapy with Albumin-bound paclitaxel and Carboplatin. Following four treatment cycles, he refused further intravenous chemotherapy and follow-up due to treatment intolerance after another two cycles. In subsequent ultrasound and CT records, the patient’s inguinal lymph nodes were found to have enlarged before chemotherapy. After chemotherapy, it decreased by 1.2 cm compared to before chemotherapy (**Table 1**, **Figure 3**).

**Figure 2:**
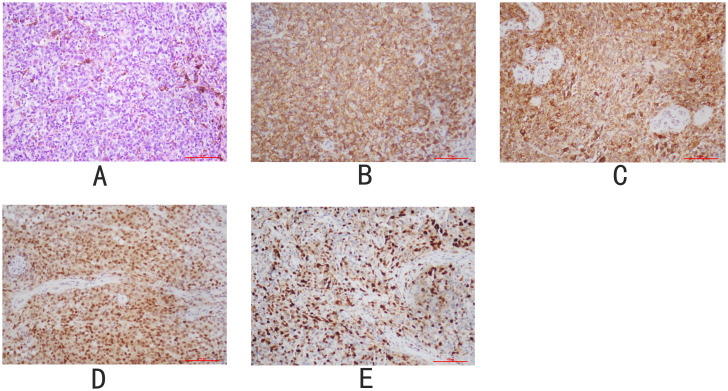
Histological manifestations of the excised specimen (Case 1). **(A)** A large number of atypical melanocytes with large, deeply stained nuclei and abundant cytoplasm. Melanocytes are arranged in clumps (hematoxylin eosin staining; Original magnification, ×200). **(B)** Tumor sections showed strong HMB-45 positive staining (magnification ×200). **(C)** Tumor sections showed strong positive Melan A staining (magnification ×200). **(D)** Tumor sections showed strong SOX-10 positive staining (magnification ×200). **(E)** The tumor section shows strong Ki67 positivity in 30% of the cells. (magnification ×200).

**Table 1:** Comparison of lymph node size before and after adjuvant therapy of Case 1.

Treatment Stage	Ultrasound (cm)		CT (cm)	
Laterality (Left/Right)	L	R	L	R
Preoperative	2.89*2.72	0.72*0.35	3.2*2.7	–
Pre-chemotherapy	5.33*2.229	3.87*1.72	3.3*2.5	–
Post-chemotherapy	2.83*0.76	2.01*0.97	2.8*1.3	–

Bilateral inguinal lymph nodes were enlarged before adjuvant therapy, and significantly reduced after adjuvant therapy.

**Figure 3:**
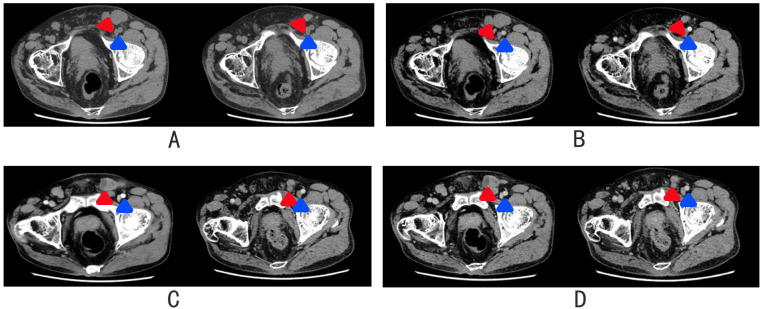
Comparison of CT manifestations before and after chemotherapy (Case 1). The image before treatment is on the left, while the image after surgery is on the right. **(A)** Comparison plain scan phase; **(B)** Comparison of arterial phase; **(C)** Comparison of venous phase; **(D)** Comparison of delayed phase. Lymph nodes are indicated by red arrows, and external iliac vessels are indicated by blue arrows.

Six months later, the patient experienced dizziness and headaches. A follow-up enhanced cranial CT scan presented a nodular lesion at the left frontal-temporal junction with well-defined borders, ring-like enhancement, and a fluid-filled area, measuring approximately 2.1 × 1.9cm (**Supplementary Figure 2**). Because of the considered brain metastasis, the previous intravenous chemotherapy regimen was continued, supplemented with stereotactic radiotherapy (SRT) subsequently. However, this treatment series did not prevent the progression of metastasis, and the patient died 18 months after diagnosis of brain metastases.

### 2.2 Case 2

The second patient was a 67-year-old male who presented with a penile mass with ulceration for five years. Biopsy from the dermatology department yielded a diagnosis of penile melanoma. Detailed physical examination also found a movable, firm mass in the left inguinal region, approximately the size of a soybean. No suspicious lesions were found on the remaining skin and mucous membranes. Inguinal ultrasound showed multiple bilateral enlarged lymph nodes. The largest node on the right measures approximately 0.69 × 0.34cm and shows no blood flow, while the largest on the left measures approximately 0.71 × 0.41cm and shows punctate blood flow. Given the possibility of distant metastasis, PET-CT was performed, which showed increased density in the anterior abdominal wall of the left inguinal region and within the pelvic cavity, with unevenly increased FDG uptake.

Following the discussion, a partial penile resection was performed under combined spinal-epidural anesthesia. The other detailed surgical process is similar to that of case 1. During postoperative pathology examination, spindle-shaped tumorous cells were distributed diffusely under the microscope, with large nuclei and oval nucleoli. Mitotic figures are frequently observed, and extensive melanin deposits are visible within the cytoplasm. The final diagnosis of primary penile melanoma was in line with the former report (**Supplementary Figure 3**). The patient left the hospital 7 days after surgery and was advised to undergo adjuvant treatment in our Department of Oncology. Following up by telephone, the patient refused adjuvant treatment. He underwent total gastrectomy for primary gastric cancer 68 months after the initial surgery and died half a year later.

### 2.3 Case 3

The third patient was a 64-year-old male who presented with a 1-year history of ulceration on the inner foreskin, which had enlarged over the past 3 months. The patient underwent penile mass excision at our hospital six years ago for penile carcinoma (Postoperative pathology indicated squamous cell carcinoma), and no adjuvant therapy was administered subsequently. We conducted a physical examination on his admission this time. The patient presented with a redundant prepuce and retractable foreskin. A single, approximately 1cm in diameter, roundish mass is observed on the dorsal aspect of the glans near the corona. An ulcerated area measuring approximately 3.0 × 3.0cm with red spots is visible on the ventral inner foreskin without foul odor, which bleeds readily on palpation. No suspicious lesions were found on the remaining skin and mucous membranes. Considering his medical history and physical examination results, a pelvic enhanced CT examination was suggested, during which an oval shadow measuring approximately 4.7 × 3.2cm is visible inferior to the left iliac artery and vein within the left pelvic wall.

Following discussion and communication with the patient, a surgical plan was made, including glans excision, circumcision, and pelvic mass resection. Following successful anesthesia and supine positioning, exploration revealed scar-like changes on the right surface of the glans penis, extending deep and firm. The lesion was excised with 0.5cm of surrounding normal tissue intact. Intraoperative pathology of the excised lesion indicated granulomatous inflammation. Then circumcision was subsequently performed, and a urinary catheter was placed. Before the third procedure of pelvic mass resection, we elevated the left lower abdomen and reapplied the routine antiseptic. An oblique incision was made in the left lower abdomen, sequentially cutting through the skin, subcutaneous tissue, and the muscle layer until reaching the extraperitoneal space, aiming to expose the left iliac vessels. A left pelvic mass was then revealed, measuring approximately 4.0 × 5.0cm, closely adherent to the left pelvic wall, iliac vessels, obturator vessels, and obturator nerve. The mass was dissected along its margins and completely excised.

Postoperative histopathological examination revealed atypical cell proliferation diffusely distributed throughout the tissue. Immunohistochemical staining of the penile mass showed CK(–), Vimentin(+), S-100(+), HMB-45(+), Melan A(+), Ki-67 (approximately 50%+)(**Supplementary Figure 4**), while that of pelvic mass presented CK (-), Vimentin (+), P63 (-), Synaptophysin (-), CD56 (-), GATA-3 (-), CK7 (-), S-100 (-), HMB-45 (+), Melan A (+), Ki-67 (approximately 50%+). These findings indicated a diagnosis of penile melanoma with pelvic metastasis. The patient left the hospital 10 days after surgery and was recommended for adjuvant treatment. After telephone follow-up, the patient refused reexamination and adjuvant treatment and died 13 months later.

### 2.4 Case summary

The three patients with penile melanoma reported herein all denied a prior history of melanoma, and no suspicious cutaneous or mucosal lesions were detected on physical examination. After multidisciplinary discussion, all patients underwent surgical treatment, and postoperative pathological examination confirmed the diagnosis of primary penile melanoma. The clinical data are summarized as follows (**Supplementary Table 1**).

## 3 Discussion

Penile melanoma (PM) is an extremely rare malignant tumor of the male genitalia (2). According to data from the U.S. National Cancer Database (SEER) from 2000 to 2018 (8), melanoma accounts for approximately 11.7% of penile non-squamous cell carcinomas, representing a relatively small proportion among all penile cancer patients. However, its prognosis is significantly poorer compared to penile squamous cell carcinoma (SCC), with a 10-year cancer-specific mortality rate as high as 38.4%—markedly higher than the 16.6% observed in matched SCC patients over the same period (9). This indicates that although penile melanoma is rare, it is highly malignant and requires particular attention in diagnosis and clinical management.

We reviewed literature published over a ten-year search period to make the summary of case features and treatments closer to the current era. Using the query (melanoma OR malignant melanoma) AND (penis OR glans penis), we identified 27 cases of penile melanoma from PubMed, EMBASE and Web of Science. All data are summarized in **Table 2**, which shows characteristics similar to our cases. Age is one of the key epidemiological characteristics of penile melanoma, which occurs primarily among middle-aged and elderly men, and the median age of diagnosis was 66.5 years old according to our review of 27 patients (10). PM can occur in various locations, appearing on the glans penis or foreskin surface, and may also involve areas such as the urethral opening, corona sulcus, and penile shaft (3, 5). Among the three patients reported in this study, two cases occurred on the glans penis, and one case occurred on the inner foreskin. In terms of etiology, unlike common skin melanoma, penile melanoma shows no clear association with sun exposure (2).

**Table 2:** Summary of all reported cases from our literature review.

Year	Reference	Age	Lesion site	Lesion presentation	Lesion size (cm)	Inguinal lymph nodes	Surgical treatment	Follow-up time(month)	Outcome	Other treatments	Chemotherapy	Immunotherapy	Breslow(mm)	IHC	Recurrence	Treatment for recurrence
2016	Turnbull (35)	50	Coronal Sulcus	Ulcer	–	–	Biopsy	2	Died	–	–	–	1.2	–	–	–
–	–	30	Glans penis	Hyperpigmentation	–	–	partial penectomy ,circumcision	–	–	–	–	–	0.1	–	–	–
–	–	40	Glans penis	Hyperpigmentation	–	–	partial penectomy ,circumcision	–	–	–	–	–	0.44	–	–	–
2017	Baraziol (18)	69	Glans penis,Foreskin	Hyperpigmentation	–	–	local excision	50	Alive	–	–	–	–	–	–	–
2017	Jabiles (36)	40	Penile shaft	Hyperpigmentation,Ulcer	–	–	local excision	221	Alive	–	–	–	3.2	–	–	–
–	–	17	Glans penis	Hyperpigmentation	–	Bilateral	local excision	–	–	–	–	–	0.38	–	–	–
–	–	39	Foreskin	Hyperpigmentation,Ulcer	–	–	local excision	38	Died	–	–	–	–	–	Bilateral Inguinal Lymph Nodes	Dacarbazine+ Interferon
–	–	58	Coronal Sulcus	Hyperpigmentation,Ulcer	–	–	local excision	221	Alive	–	–	–	0.75	–	–	–
–	–	53	Foreskin	Hyperpigmentation,Ulcer	–	Bilateral	circumcision	63	Alive	–	–	–	4.7	–	Local Recurrence	Local Excision
–	–	60	Coronal Sulcus	Hyperpigmentation,Ulcer	–	–	partial penectomy	6	Alive	–	–	–	1.6	–	–	–
2018	Tokita (37)	65	Urethral Opening	Mass	–	Left	partial penectomy	20	Alive	–	Dacarbazine,Nimustine,Vincristine	Nivolumab,Interferon	–	–	–	–
2018	Maruyama (38)	71	Foreskin	Hyperpigmentation,Ulcer	–	–	circumcision	24	Alive	–	–	Topical Interferon	11	–	–	–
2019	Aoki (39)	77	Glans penis,Urethral Opening	Hyperpigmentation,Mass	60,5	–	Penectomy	6	Alive	–	–	–	2.1	HMB-45,SOX-10,Melan A	–	–
2020	Beaudoux (34)	69	Coronal Sulcus	Hyperpigmentation,Nodule	1.2*0.8	–	–	32	Alive	–	–	nivolumab	–	–	–	–
2020	O’Hern (40)	69	Penile shaft	Hyperpigmentation,Ulcer	2.5*2.3	–	local excision	20	Alive	Tretinoin Cream,Imiquimod Cream 5%	–	–	1.76	Melan A,SOX-10	–	–
2020	Wincewicz (41)	90	Glans penis	Hyperpigmentation,Ulcer	3.5	–	–	–	–	–	–	–	12.5	HMB-45	–	–
2021	Burity (42)	79	Glans penis	Hyperpigmentation	1.4	–	partial penectomy	24	Alive	–	–	–	55	S-100,HMB45,Melan A	–	–
2022	Kaakoua (3)	70	Glans penis ,rethral Opening	Hyperpigmentation,Nodule,Ulcer	1	Right	–	24	Alive	–	–	pembrolizumab	4	–	–	–
2023	Pósfai (2)	62	Urethral Opening	Hyperpigmentation	2.9	Right	local excision	–	–	–	–	Ipilimumab,nivolumab	5.837	HMB-45,SOX-10,PRAMK	–	–
–	–	68	Foreskin	Hyperpigmentation	3	–	circumcision	–	–	–	–	–	1	SOX-10,PRAMK	–	–
2023	Kennedy (43)	76	Foreskin	Hyperpigmentation	2.2*0.7	–	circumcision	–	–	–	–	–	–	–	–	–
2023	Castro (44)	28	Glans penis	Hyperpigmentation	0.8	–	local excision	12	Alive	–	–	–	–	–	–	–
2024	Kumar (23)	38	Glans penis	Hyperpigmentation	–	–	partial penectomy	12	Alive	–	Cisplatin,Bleomycin,Vincristine	Thymosin	–	HMB45,S-100	–	–
2024	Villa-Gonzalez (45)	86	Glans penis	Hyperpigmentation	3.1	–	partial penectomy	120	Alive	–	–	–	0.6	–	–	–
–	–	73	Glans penis	Hyperpigmentation	2	–	partial penectomy	72	Died	–	–	–	0.6	–	–	–
–	–	75	Glans penis	Hyperpigmentation	Glans Penis	–	partial penectomy	5	Alive	–	–	–	0.6	–	–	–
2026	Guarda (46)	47	Glans penis	Hyperpigmentation,Nodule,Ulcer	Glans Penis	Bilateral	Penectomy	12	Died	–	–	–	12	–	–	–

Currently, due to the rarity of PM, it lacks available screening methods and early diagnostic criteria. Initial diagnosis primarily relies on physical examination and a survey of medical history. Early manifestations of PM commonly include irregular pigmented patches or nodules, sometimes accompanied by surface ulceration or exudation, accounting for 30.7% among the 27 patients in our review. Subsequent definitive diagnosis and staging rely on imaging techniques and histopathology. Enhanced CT is important in invasion/metastasis assessment, disease staging, and treatment planning. Due to its exceptional soft tissue resolution, MRI can also distinguish nearby invasions such as corpus cavernosum, thereby facilitating local staging (11). As a highly accurate method, PET-CT demonstrates its sensitivity in detecting metastasis, providing evidence for the decision-making of treatments. Additionally, the evaluation of lymph node metastasis has been enhanced by cutting-edge techniques that combine sentinel lymph node biopsy with ultrasound and nuclear medicine imaging. The use of tracers like 99mTc-labeled nanocolloids along with fluorescent dyes (e.g., ICG) allows for precise metastasis localization, thereby reducing the trauma and complications of traditional lymph node dissection (12, 13).

Pathological examination remains the gold standard for confirming the diagnosis and staging. Immunohistochemical staining for markers such as S-100 protein, HMB-45, and Melan-A serves as a crucial tool for diagnosing and differentiating melanoma, of which the combination effectively enhances diagnostic accuracy (14). Currently, there is no specialized staging system for primary penile melanoma, and the main reference is to cutaneous melanoma (15); the three cases we reported also referenced this staging system. However, penile melanoma belongs to the category of mucosal melanoma and exhibits significant differences from common cutaneous ones, leading to the AJCC system not being fully applicable (16).

In terms of differential diagnosis, it must be clinically differentiated from pigmented nevi, inflammatory lesions, and lichen sclerosus. Penile lichen sclerosus is a chronic inflammatory skin disease that is commonly found on the male genitalia. Its chronic inflammatory state may induce abnormal proliferation of local melanocytes, potentially progressing to melanoma. A study reported 11 PM cases, among which 9 of them showed pathological evidence of lichen sclerosus, suggesting a potential causal relationship that may continuously stimulate melanocytes and promote their malignant transformation under a chronic inflammatory environment (17). This potential causality may also be supported by our reported cases indirectly. Patient 1 presented with phimosis, while lichen sclerosus is one of the most common causes that trigger it. Patient 3 had a history of penile squamous cell carcinoma, redundant prepuce, and ulcer signs during physical examination. Both patients have a highly associated history with lichen sclerosus, and likely to be fully exposed to chronic inflammation. We therefore infer that these two patients likely had undiagnosed and untreated lichen sclerosus before presentation, which may be responsible for the development of primary melanoma. Notably, Patient 3 did not undergo circumcision during his prior treatment for squamous cell carcinoma. Had synchronous circumcision been performed at that time, his subsequent risk of developing penile melanoma could have been significantly lowered by reducing chronic inflammatory stimuli. Therefore, his experience also serves as a warning for the surgeries of other penile malignancies. Although the growth site of PM is easy to detect, patients often avoid seeking medical help in the early stages. Therefore, the diagnosis is often made in late stages, which may be because pigmentation is a common symptom that patients do not pay attention to, or it may be because the affected area is private (18–20).

As a rare malignant tumor, primary treatment relies on local surgical excision, particularly for those early localized lesions. For larger lesions, partial penile resection becomes a more reasonable option to guarantee adequate tumor control, especially for those lesions involving deep penile structures. A comprehensive treatment combining partial resection with lymph node dissection has been reported for late-stage or locally advanced lesions, yet its efficacy needs further verification (10). According to our review, among all the reported patients, 23 underwent surgical treatment, one underwent biopsy only, and another three declined surgery due to the sensitive location. Of those treated surgically, 12 underwent local excision (four patients with tumors only growing on the foreskin underwent circumcision), nine underwent partial penile resection (two combined with circumcision), and two underwent radical penile resection due to its extensive infiltration. To compare the prognoses of local excision and partial penile resection, we conducted a preliminary survival analysis of the two surgical methods (21 cases in total). The survival analysis results showed no significant difference in prognosis (**Supplementary Figure 5**). However, due to the limited number of pathological cases included and their varying tumor extent and location, the value of our non-significant result is limited, as no statistical equivalence can be established. However, this descriptive summary still suggests a common surgical strategy that surgical plans should be personalized based on the affected area and the principle of thorough clearance, rather than simply choosing one approach based on any prognostic difference.

Based on the surgical experience of three patients in our center, we conclude that although penile melanoma, penile squamous cell carcinoma, and benign penile proliferative lesions differ significantly in biological behavior and invasiveness, their surgical strategies can be mutually referenced due to the similar predilection sites and lesion sizes. Local extended excision is recommended for localized lesions, while partial or total penectomy is selected for larger tumors according to the depth of invasion. Meanwhile, attention should be paid to preserving organ function and achieving negative surgical margins during surgery. Due to the rarity and poor prognosis of penile melanoma, even if the surgical treatment principles are similar, multidisciplinary discussions with dermatologists and oncologists after knowing the pathological results will be a favorable strategy for prognosis and further therapies.

As penile melanoma is highly invasive, the tumor rapidly spreads locally and is prone to lymph node metastasis, normally in the inguinal zone. Traditionally, Inguinal lymph node dissection (ILND) is the gold standard for diagnosing and treating lymph node metastasis. However, it carries a high rate of perioperative complications, including lymphatic leakage, lymphatic cysts, wound infections, and lymphedema, which significantly impact patients’ quality of life and postoperative recovery (21, 22). Current literature recommends prophylactic superficial inguinal lymph node dissection for patients with tumor thickness exceeding 1.5mm, while also providing a basis for subsequent treatment and pathological staging (23–25).

Radiation therapy is commonly used for adjuvant therapy following surgery, local recurrence, and advanced cases where surgery is not feasible (6). However, due to the unique anatomical structure of penile tissue and the pathological characteristics and biological behavior of penile melanoma, which determine its low sensitivity to radiotherapy, it is difficult to achieve the desired therapeutic effect. Therefore, chemotherapy is sometimes applied. Bevacizumab and a combination of cabozantinib with paclitaxel exhibit certain efficacy in treating urogenital melanoma in randomized controlled trials (26, 27). Our first case achieved some therapeutic effect of chemotherapy following surgery and remained uneventful in prognosis under a combination of chemotherapy and radiotherapy after developing brain metastases, suggesting the potential effectiveness of these two adjuvant therapies.

In addition to traditional non-surgical therapies such as radiotherapy and chemotherapy, some novel therapies have been recently used for the treatment of malignancies. For example, immune checkpoint inhibitors (ICIs) have garnered significant attention, especially for situations when traditional treatments turn ineffective and bring new hope for PM management, in which therapy targeting PD-L1 and CTLA-4 serves as an important target. PD-1 is overexpressed in mucosal melanoma, which provides a foundation for the application in penile melanoma (28). Due to its rarity, even though clinical cases are limited, there have been some reports suggesting the potential efficacy (3, 23). A retrospective study also demonstrated that combination therapy of anti-PD-1 and anti-CTLA-4 drugs is a feasible approach for downstaging before surgery, which provides new insights for neoadjuvant therapy (29). Recently, specific genetic mutations such as BRAF and KIT have gradually emerged as key research targets in the immunotherapies of mucosal melanoma, of which overall data suggest that KIT mutations are relatively common in certain sites, while BRAF mutations are comparatively less frequent (30, 31). In penile melanoma, the low prevalence of BRAF mutations limits its applicability. In contrast, imatinib, as a targeted inhibitor of KIT mutations, demonstrates therapeutic efficacy against KIT mutation-positive mucosal melanoma (32–34). However, KIT testing was not performed for Case 1 due to the limited available sample, which may limit the assessment of the patient’s prognosis. Additionally, gene mutations in TSC1, mTOR, POLE, and ATRX are also revealed at high frequencies in mucosal melanoma, suggesting that future novel therapeutic options (31).

## 4 Conclusion

In summary, penile melanoma represents a highly aggressive malignancy, and no sufficient summary and unified consensus on its characteristics and treatment have been formed due to its rarity. Through a detailed presentation of three cases and a literature review, this study consolidates current knowledge on its symptom characteristics, diagnostic challenges, and therapeutic options. Our analysis reaffirms that complete surgical excision remains the key to treatment, with emerging adjuvant and neoadjuvant strategies, particularly in advanced or refractory situations. Given the anatomical and psychosocial significance of the affected site, decision makers must balance oncological efficacy with life quality preservation. Further collaborative research and standardized case reports are still essential in the future to refine staging systems, validate novel therapies, and ultimately improve outcomes for patients with this uncommon but serious disease.

## Data Availability

The original contributions presented in the study are included in the article/**Supplementary Material**. Further inquiries can be directed to the corresponding authors.
