# Supplementary material for: Case Report: Penile malignant melanoma: insights from a three-case series and literature review on diagnosis and management
Source: Front Oncol. 2026 Jul 17;16:1817540. doi: 10.3389/fonc.2026.1817540 (PMC13423700; doi:10.3389/fonc.2026.1817540)
Supplement: Supplementary file 1 [file DataSheet1.zip › Supplementary Files/Supplementary Files.docx]

**
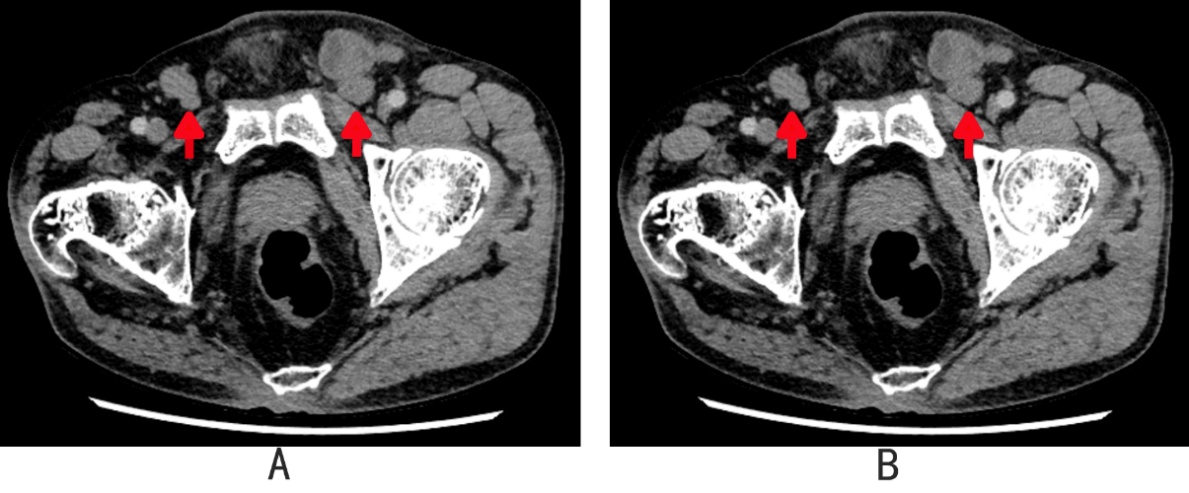
**

**Supplementary Figure 1. Preoperative CT images of Case 1.** Multiple enlarged lymph nodes were shown in bilateral inguinal zone with enhancement during the arterial phase (A) and the venous phase (B).

**
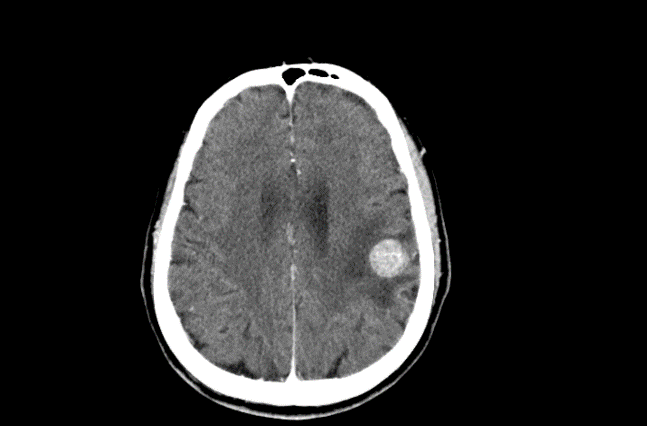
**

**Supplementary Figure 2. Enhanced Cranial CT scan 6 months after surgery of Case 1.** A 2.1*1.9cm nodular high-density shadow was seen at the left frontotemporal junction

**
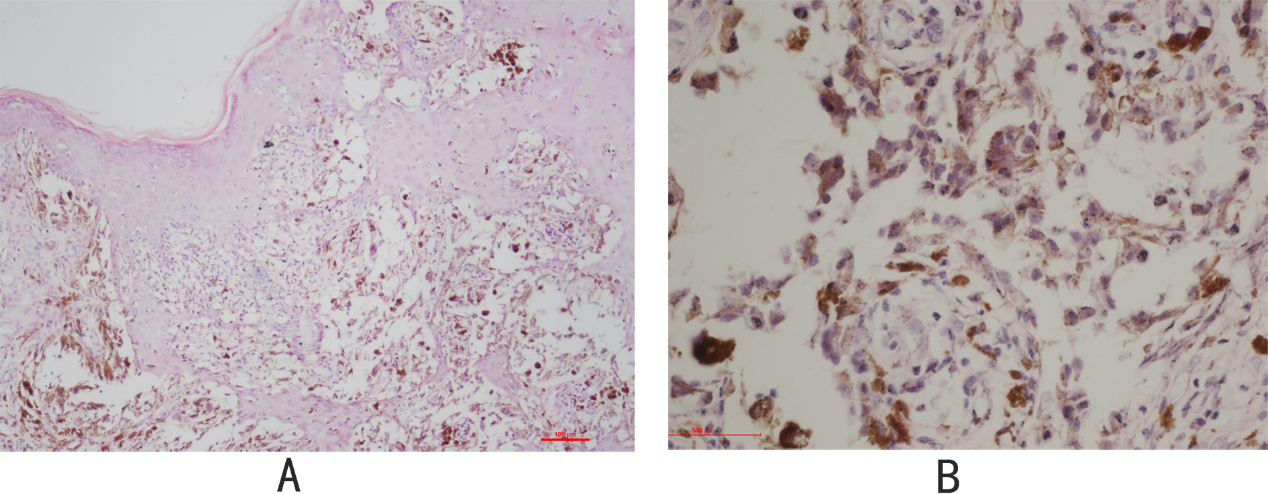
**

**Supplementary Figure 3. Histological manifestations of excised specimens of Case 2. A** The tumor cells exhibit a spindle-shaped morphology with diffuse distribution. They possess large oval nuclei, distinct nucleoli, and frequent mitotic figures. Abundant melanin deposits are observed within the cytoplasm (hematoxylin eosin staining; Original magnification, ×200). B High-power microscopic view of tumor cells.

**
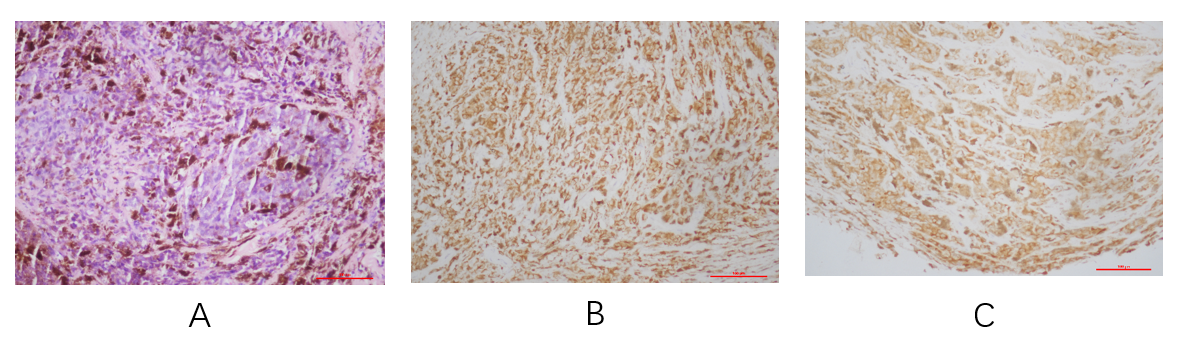
**

**Supplementary Figure 4. Histological manifestations of excised specimens of Case 3.** (A) Atypical cell hyperplasia is observed within the tissue, showing a diffuse distribution (hematoxylin eosin staining; Original magnification, ×200). (B) Tumor sections showed strong HMB-45 positive staining (magnification ×200). (C) Tumor sections showed strong positive Melan A staining (magnification ×200).

**
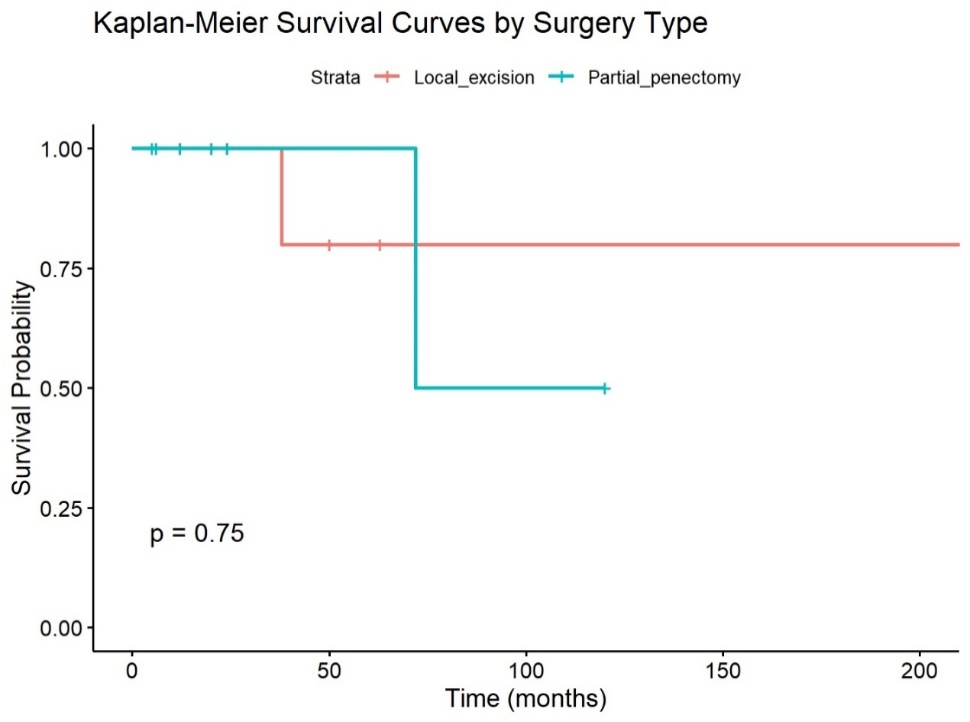
**

**Supplementary Figure 5. Survival analysis between different surgical protocols.** The overall survival between local excision and partial penectomy shows no significant difference.

**Supplementary Figure 1: Basic information of the three patients**

| age | Lesion site | Lesion Size (cm) | IHC | Breslow (mm) | margin distance(cm) | Inguinal lymph nodes | Treatments | Follow-up Time (month) | Outcome | cause of death |
| --- | --- | --- | --- | --- | --- | --- | --- | --- | --- | --- |
| 46 | Glans penis | 2*2 | CK(±)，Vimentin(+)，HMB-45(+)，Melan A(+)，S-100(+)，SOX-10(+)，Ki-67(>30 %) | 2.3 | 3 | Bilateral | partial penectomy+chemotherapy | 24 | died | brain metastasis |
| 67 | Glans penis | 1.5*1.5 | - | 1.5 | 2 | Bilateral | partial penectomy | 74 | died | other causes |
| 64 | Foreskin | 1*1 | CK(-) Vimentin(+) S-100(+) HMB-45(+) Melan A(+) Ki-67(约50%+) B3:CK(-) Vimentin(+) P63(-) Synaptophysin(-) CD56(-) GATA-3(-) CK7(-) S-100(+) HMB-45(+) Melan A(+) Ki-67(约50%+) | 1.9 | 0.5 | Bilateral | partial penectomy+circumcision | 13 | died | disease-related death |
| IHC：Immunohistochemistry；margin distance：It refers to the tissue distance from the tumor edge to the surgical margin in the resected specimen. | | | | | | | | | | |
