# Supplementary figures and images for: Case Report: Penile malignant melanoma: insights from a three-case series and literature review on diagnosis and management
Source: Front Oncol. 2026 Jul 17;16:1817540. doi: 10.3389/fonc.2026.1817540 (PMC13423700; doi:10.3389/fonc.2026.1817540)

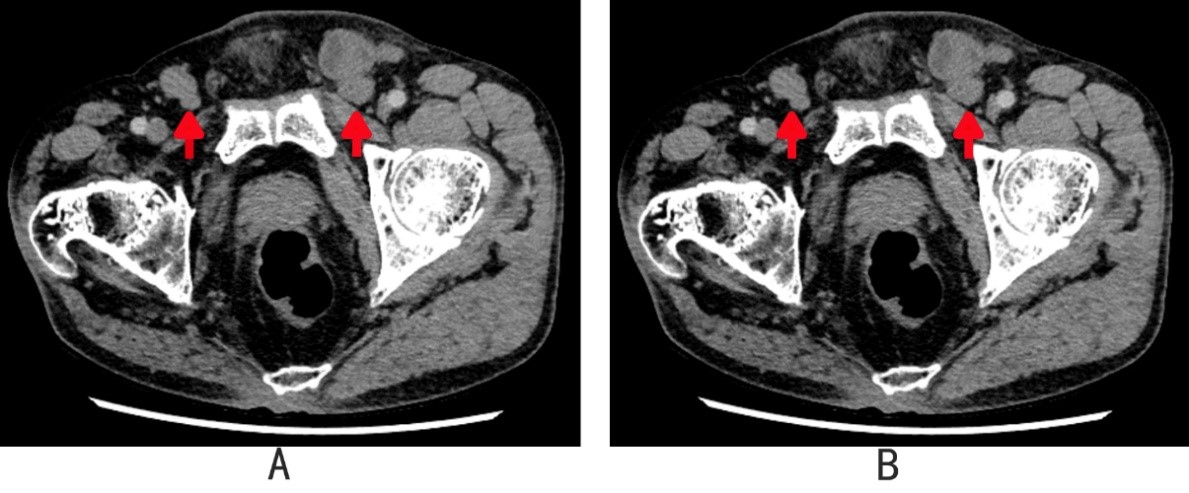

Supplement: Supplementary file 1 [file DataSheet1.zip › Supplementary Files/Supplementary Figure 1.tiff]

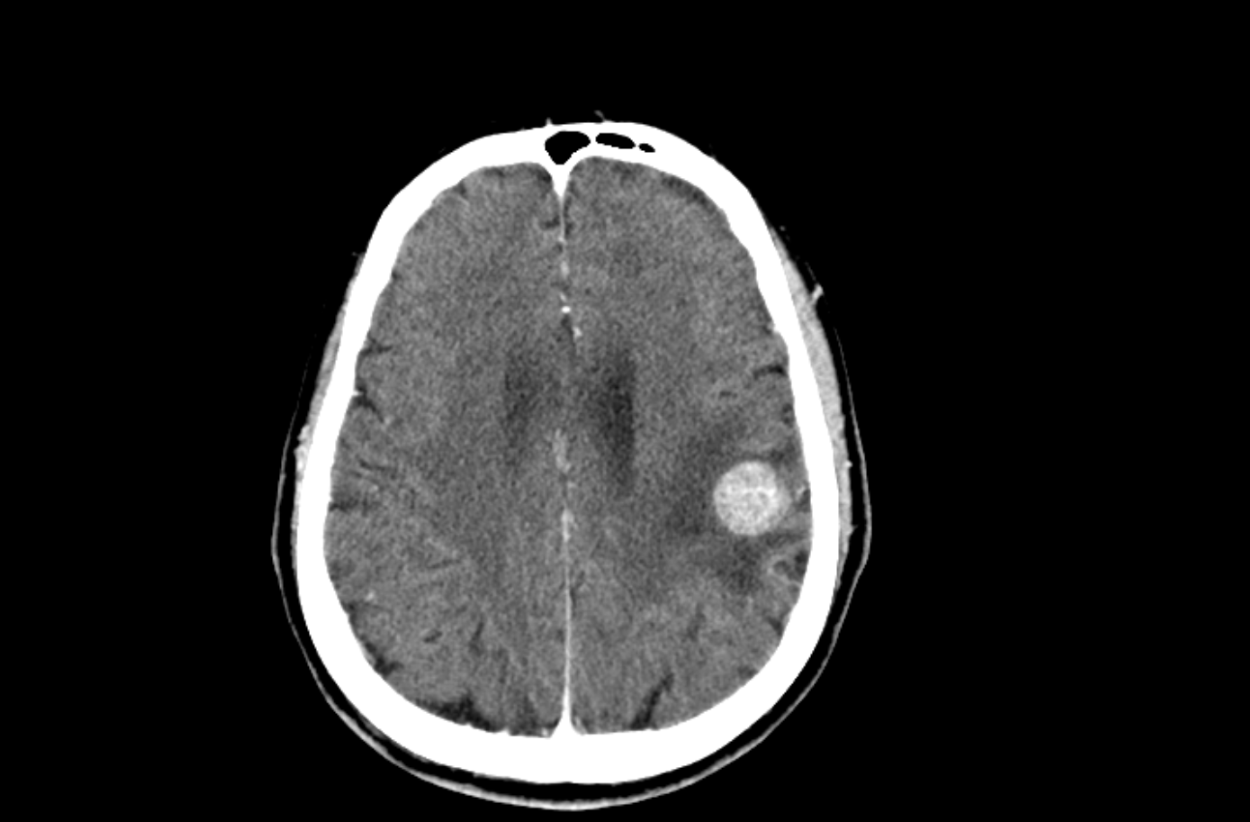

Supplement: Supplementary file 1 [file DataSheet1.zip › Supplementary Files/Supplementary Figure 2.tiff]

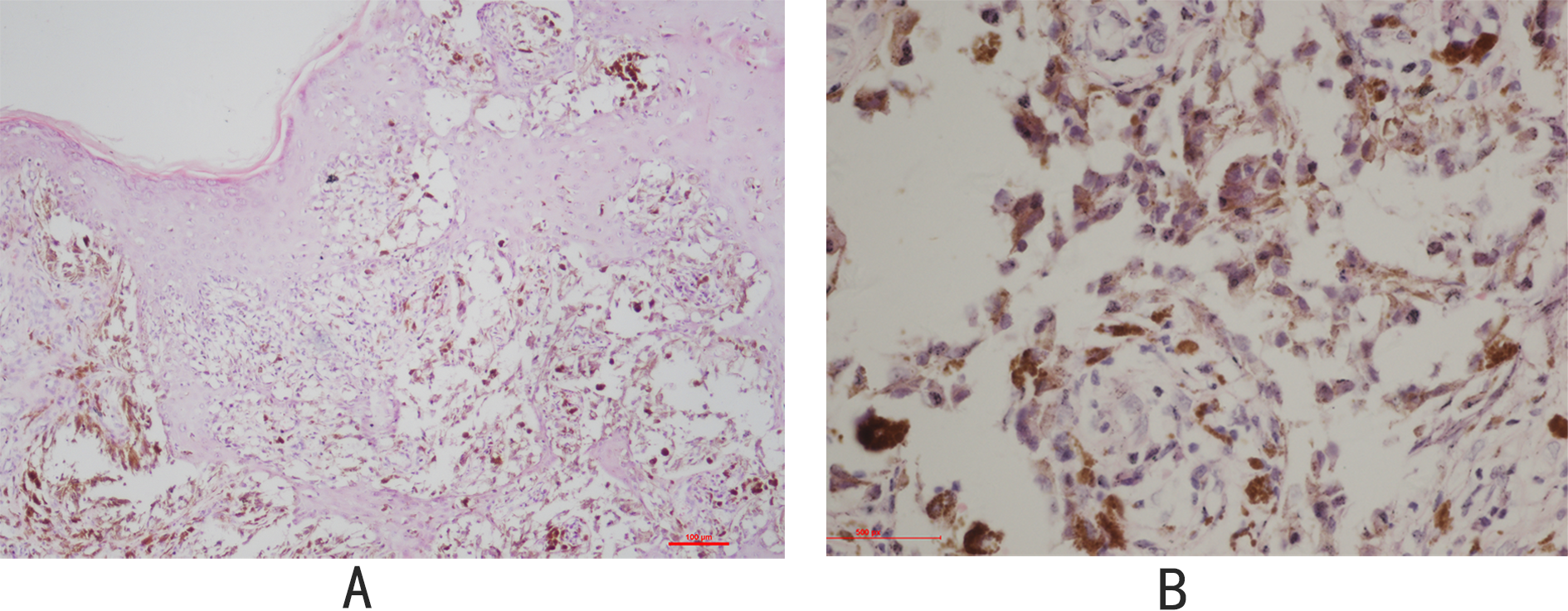

Supplement: Supplementary file 1 [file DataSheet1.zip › Supplementary Files/Supplementary Figure 3.tif]

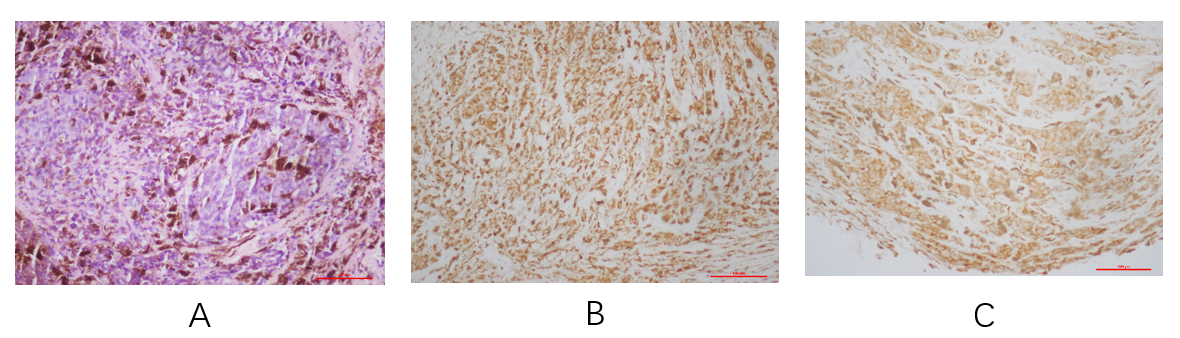

Supplement: Supplementary file 1 [file DataSheet1.zip › Supplementary Files/Supplementary Figure 4.tiff]

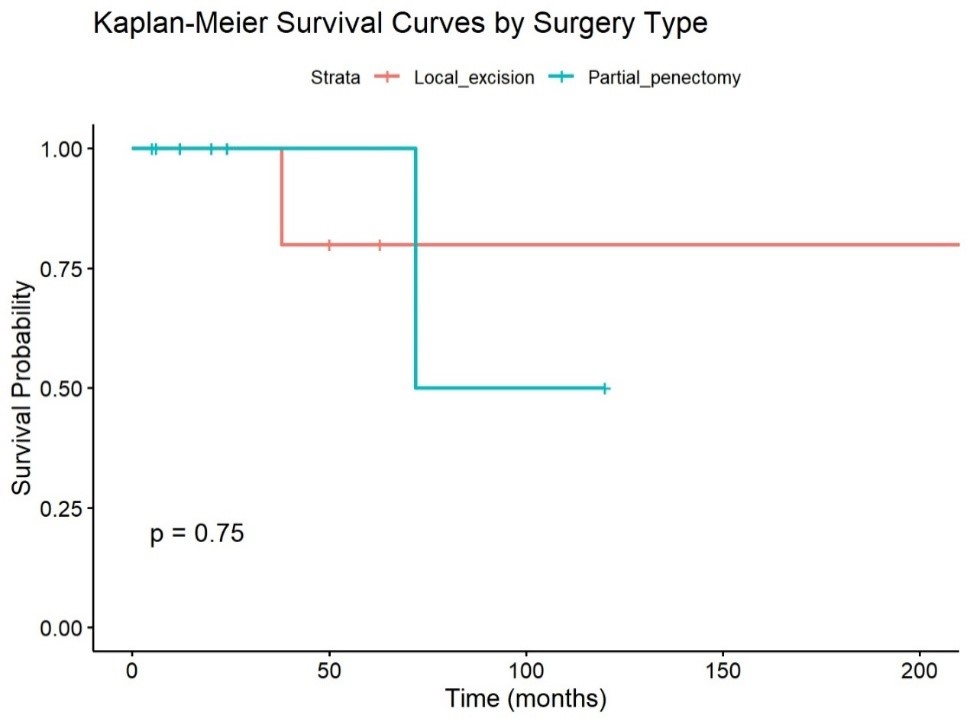

Supplement: Supplementary file 1 [file DataSheet1.zip › Supplementary Files/Supplementary Figure 5.tiff]
